# Characterization and Phylogenetic Analysis of a Novel GH43 β-Xylosidase From *Neocallimastix californiae*

**DOI:** 10.3389/ffunb.2021.692804

**Published:** 2021-07-07

**Authors:** Marcus Stabel, Julia Hagemeister, Zacharias Heck, Habibu Aliyu, Katrin Ochsenreither

**Affiliations:** Process Engineering in Life Sciences 2: Technical Biology, Karlsruhe Institute of Technology, Karlsruhe, Germany

**Keywords:** *Neocallimastigomycota*, anaerobic fungi, lignocellulose, xylan degradation, xylanase

## Abstract

Degradation of lignocellulosic materials to release fermentable mono- and disaccharides is a decisive step toward a sustainable bio-based economy, thereby increasing the demand of robust and highly active lignocellulolytic enzymes. Anaerobic fungi of the phylum *Neocallimastigomycota* are potent biomass degraders harboring a huge variety of such enzymes. Compared to cellulose, hemicellulose degradation has received much less attention; therefore, the focus of this study has been the enzymatic xylan degradation of anaerobic fungi as these organisms produce some of the most effective known hydrolytic enzymes. We report the heterologous expression of a GH43 xylosidase, Xyl43Nc, and a GH11 endoxylanase, X11Nc, from the anaerobic fungus *Neocallimastix californiae* in *Escherichia coli*. The enzymes were identified by screening of the putative proteome. Xyl43Nc was highly active against 4-Nitrophenol-xylopyranosides with a K_m_ of 0.72 mM, a k_cat_ of 29.28 s^−1^, a temperature optimum of 32°C and a pH optimum of 6. When combined, Xyl43Nc and X11Nc released xylose from beechwood xylan and arabinoxylan from wheat. Phylogenetic analysis revealed that Xyl43Nc shares common ancestry with enzymes from *Spirochaetes* and groups separately from Ascomycete sequences in our phylogeny, highlighting the importance of horizontal gene transfer in the evolution of the anaerobic fungi.

## Introduction

The conversion of lignocellulose containing material into fermentable sugars is an important step toward a CO_2_ neutral economy and society (Bundeministerium für Bildung und Forschung, 2010). However, microbial accessibility of and sugar release from lignocellulosic materials requires pretreatment, which tends to be expensive, energy intensive and commonly involves the use of hazardous and toxic compounds (Rahmati et al., 2020). In contrast, biological pretreatment involves mild and eco-friendly conditions with the drawback of being time consuming and the used enzymes being expensive.

**Figure 1:**
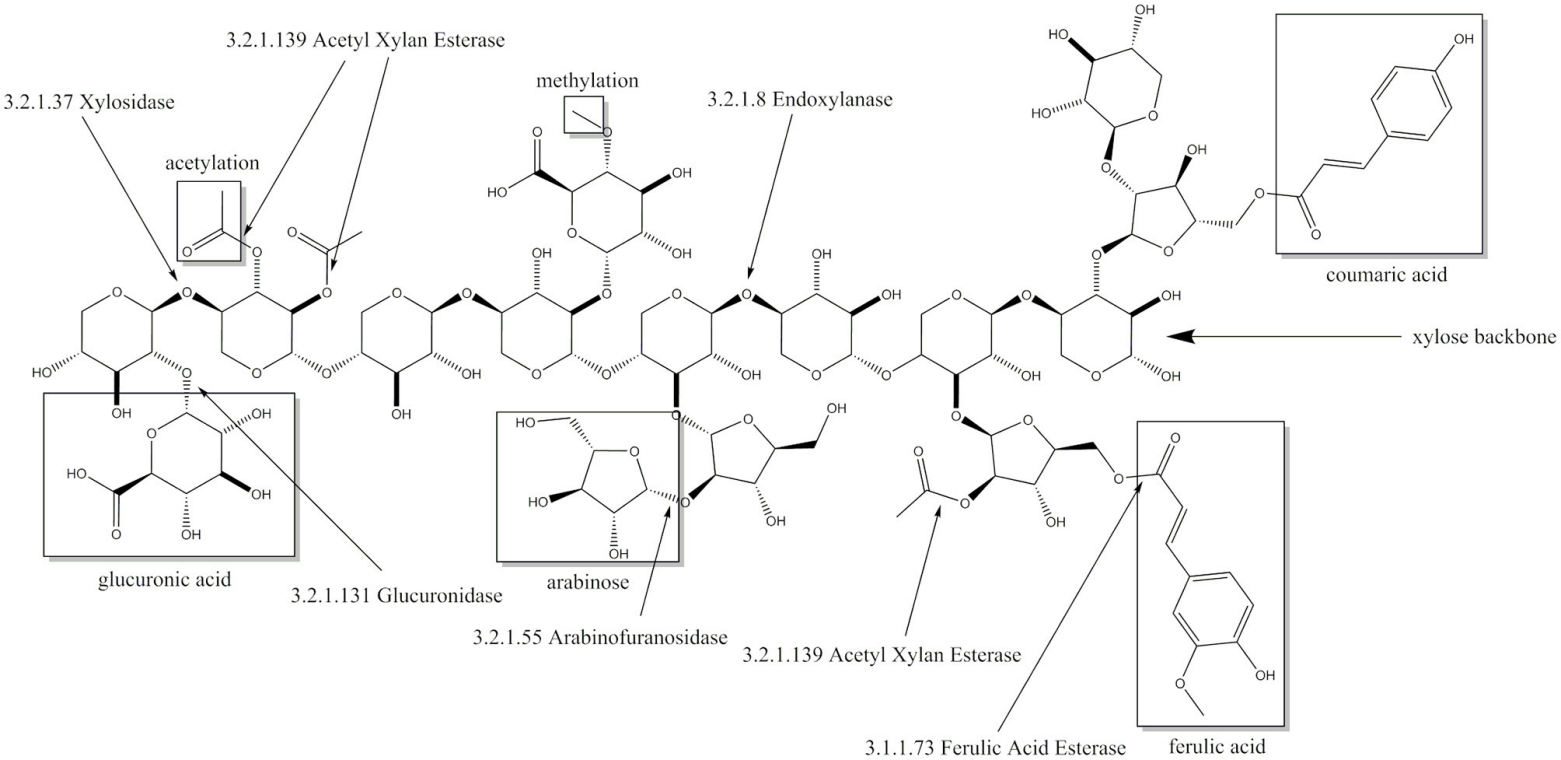
Xylose backbone of xylan with possible substitutions and the cleaving sites of corresponding enzymes including enzyme commission number.

Although hemicellulose make up around 30% of the lignocellulose dry weight (Rahmati et al., 2020), xylose is far less used as an renewable carbon source, because many of the biotechnological relevant microorganisms are unable to break down hemicelluloses such as xylans or to metabolize xylose (Horlamus et al., 2019). Xylan is the most common polymer of the hemicellulose fraction with its backbone constituting a chain of β-1,4-linked xylopyranose molecules with different possible substitutions (Figure 1). The latter include acetylations, methylations and side chains of glucuronic acid, arabinose, coumaric acid and ferulic acid. The exact composition of these substitutions varies from plant to plant and thereby requires different enzymes for each type (Biely et al., 2016). The basic enzymes which are always required are xylanases and xylosidases. Xylanases hydrolyze the backbone releasing xylose polymers which are further depolymerized to xylose by xylosidases. Substitutions of the xylose backbone can inhibit xylanase activity and thereby the whole process (Pollet et al., 2010). In this case, debranching enzymes like arabinofuranosidases, glucuronidases and acetyl xylan esterases are required (Biely et al., 2016), which remove the respective substitutions and thereby enable endoxylanase activity. In addition to their application in the degradation of lignocellulose, xylanolytic enzymes can be used in animal feed, food industry, paper production and preparation of textile fibers (Polizeli et al., 2005).

Within the fungal kingdom anaerobic fungi of the phylum *Neocallimastigomycota* have been recognized for their highly efficient lignocellulose degradation abilities (Solomon et al., 2016; Seppälä et al., 2017). *Neocallimastigomycota* are native to the digestive system of a huge variety of herbivores, of which a good overview is given by Gruninger et al. (2014). Horizontal gene transfer has been an important driver in the evolution of anaerobic fungal carbohydrate active enzymes (CAZymes) with over 50% of some glycoside hydrolase (GH) families being of non-fungal origin (Murphy et al., 2019). Although several studies have highlighted the potential of anaerobic fungi for biotechnology (Gruninger et al., 2014; Haitjema et al., 2014; Seppälä et al., 2017; Cheng et al., 2018; Hooker et al., 2019; Podolsky et al., 2019), only limited information about characterized CAZymes from these fungi has been published so far. However, some of the few characterized enzymes have shown promising properties like multiple hydrolase activities (Pai et al., 2010; Morrison et al., 2016a) and competitiveness with commercial mixtures in terms of activity (Morrison et al., 2016b). Despite these promising properties for lignocellulose degradation, the knowledge about *Neocallimastigomycota* enzymes at a biochemical level is very limited. For example, despite several having been identified just one enzyme with xylosidase activity has been fully characterized (Morrison et al., 2016a). These potential xylosidases include members of the GH family 43 (Youssef et al., 2013; Couger et al., 2015; Henske et al., 2017; Hagen et al., 2020). GH43 is one of the most prominent families containing xylosidases showing some of the highest reported xylosidase activities so far (Jordan et al., 2013, 2016; Falck et al., 2016). The family is further divided in 37 subfamilies (GH 43_1-GH 43_37) (Mewis et al., 2016) and encompasses enzymes with dual xylosidase and arabinofuranosidase activity (Huy et al., 2013; Yang et al., 2014; Zhang et al., 2019). Here we report the heterologous expression, purification and characterization of a novel GH43 xylosidase from *Neocallimastix californiae*.

## Materials and Methods

### *In silico* Characterization and Phylogenetic Analyses

A putative proteome from *N. californiae* was downloaded from GenBank (assembly accession: GCA_002104975.1). The totality of known xylosidases (EC 3.2.1.37) and endo-xylanases (EC 3.2.1.8) was downloaded from SwissProt (05.04.2018). From each dataset a local database was created in BioEdit (V. 7.0.5.3) (Hall, 1999). The respective enzyme databases were searched against the *N. californiae* database using the local BLASTp function of BioEdit. The proteome sequence with the highest similarity scores to the local xylosidase (ORY16049) and the local endoxylanase (ORY50654) databases was selected for cloning. The xylosidase and the endoxylanase were further validated via NCBI conserved domain search (Marchler-Bauer et al., 2017). Phyre2 (Kelley et al., 2015) was used for additional homology search of the xylosidase including the tertiary structure.

To perform phylogenetic analysis of Xyl43Nc, the protein was queried against the UniProtKB database (03.02.2021) using blastp. The top 100 sequences with the highest similarity score were downloaded, annotated using dbCAN (Yin et al., 2012; Zhang et al., 2018) and aligned using T-Coffee (Notredame et al., 2000). Phylogenies were reconstructed using IQ-TREE v2.0.3 (Minh et al., 2020) with model selection (Kalyaanamoorthy et al., 2017) and 1,000 ultrafast bootstraps (Hoang et al., 2017). The resulting tree was rooted using MAD (Tria et al., 2017). Tree visualization and editing was done with Evolview V3 (Subramanian et al., 2019).

### Cloning Procedure

Genes were codon optimized and synthesized by GenScript. Cloning into pET21b (Novagen) was performed using the NEBuilder® HiFi DNA Assembly Cloning Kit following the manufacturer's instructions. Colonies were picked and cultured overnight at 37°C and 120 rpm in LB-Media containing 10 g/l NaCl, 10 g/l tryptone, 5 g/l yeast extract and 50 mg/l ampicillin. Plasmids were extracted from the overnight culture using the Monarch® Plasmid Miniprep Kit from New England Biolabs following the manufacturer's instructions and correctness of the construct was confirmed by sequencing (GATC-Eurofins Genomics). pET21b constructs were transformed into CaCl_2_ competent *E. coli* BL21 pLysS (DE3) cells by heat shock transformation.

### Heterologous Enzyme Expression and Purification

TB-Medium was prepared by dissolving 12 g Trypton, 24 g Yeast extract and 4 ml 100% glycerol in 900 ml ddH_2_O. One Hundred milliliters of a sterile solution containing 0.17 M KH_2_PO_4_ and 0.72 M K_2_HPO_4_ was added to TB medium after sterilization by autoclaving. One Hundred milliliters TB-Medium was inoculated to an OD600 of 0.1 with an overnight culture and grown at 37°C and 130 rpm until reaching an OD600 of 0.6. To induce heterologous expression, IPTG was added to a final concentration of 1 mM. Subsequently, the culture was incubated overnight at 20 °C and 130 rpm. Cells were centrifuged at 4°C and 4,000 g for 10 min. The supernatant was discarded and the cell pellet was resuspended in 20 ml Binding Buffer (50 mM NaPP, 0.5 M NaCl, 50 mM Imidazol, pH 6.9). The following steps were performed at 4 °C or on ice: Cells were disrupted by ultrasonification with a 20 kHz ultrasonic homogenizer Sonopuls HD 3100 equipped with the probe MS 72 (Bandelin electronic GmbH & Co. KG) at an amplitude of 60% for 5 min with intervals of 20 s pulse and 30 s pause. Cell debris was removed by centrifugation for 1 h at 4°C and 100.000 g. The obtained protein crude extract was applied to a HIS Trap HP Column 1 ml from GE Healthcare in a ÄKTA Start System. The target protein was eluted from the column with 70% Binding Buffer and 30% Elution Buffer (50 mM NaPP, 0.5 M NaCl, 0.5 M Imidazol, pH 6.9). Obtained fractions containing the target protein were pooled, concentrated with Amicon® Ultra 15 mL Centrifugal Filters (MW 10.000) and further purified by using a HiPrep Sephacryl S-200 HR Column from GE Healthcare in the same ÄKTA system as mentioned above with 0.15 M NaCl, 50 mM NaPP pH 7 as elution buffer. The resulting fraction was concentrated using the same filters described above. Purity of the enzyme was confirmed by SDS-PAGE with a 12.5% Gel stained with coomassie stain containing 20% (v/v) methanol, 10% (v/v) acetic acid and 1 g/l brilliant blue R250 (Carl Roth). Bluestar Protein Ladder from Nippon Genetics (10–180 kDa range) was used as marker.

### Activity Assays

Activity of β-xylosidase was quantified by reactions with 500 μl 7.5 mM 4-Nitrophenol-xylopyranosides (Megazyme) in 50 mM citrate buffer (pH 5-6) or 50 mM natrium phosphate buffer (pH 6-7.5) as substrate. The reaction was started by adding 5 μl of enzyme with a concentration of 0.3–0.5 mg/ml to the substrate solution. Exact enzyme concentrations were determined using ROTI®Quant universal (Carl Roth) following the manual instructions. The reactions were stopped after 2 min with 1.5 M Na_2_CO_3_. All reactions were performed in triplicate. Absorption was measured at 405 nm in 96-well plates using Epoch Microplate Spectrophotometer (BioTek Instruments GmbH) and product concentration was calculated based on a calibration curve (linear range between 0 and 0.2 mM). Distribution of samples to the 96-well plate was performed by a Brand Liquid Handling Station.

For determining influences of different ions on the reaction, 1 mM or 10 mM of BaCl_2_, MgCl_2_, MnCl_2_, NiCl_2_, KCl, LiCl, ZnCl_2_, CaCl_2_, CoCl_2_ or CuCl_2_ were added prior to the substrate solution.

For temperature stability assays, enzyme solutions were incubated at different temperatures for 1 h and then applied to the activity assay as described above.

For determination of kinetic parameters, substrate concentrations of 0.25–7.5 mM 4-Nitrophenol-xylopyranosides were used. For product inhibition tests xylose concentrations of 0–10 mM were used. The reaction time for the activity assay with 4-Nitrophenolarabinofuranosides (Megazyme) was increased to 4 min due to relatively lower activity. The calculation of K_m_, A_max_ and K_i_ was done by curve fitting with the Enzyme Kinetics add-on (V 1.10) in Origin (OriginLab V. 9.65).

For testing the β-xylosidase under more natural conditions an endoxylanase was needed and identified as described above. The endoxylanase temperature optimum was determined in reactions of 5 g/l purified beechwood xylan in 50 mM NaPP pH 6 as substrate at temperatures between 30 and 60°C. The reactions were started by adding 5 μl purified enzyme (0.3–0.5 mg/ml) to 500 μl of pre-heated substrate. After 1 h, a sample was taken for analysis using 3,5-dinitrosalicylic acid (DNS) assay adapted from McCleary and McGeough (2015), which also stopped the reaction. Specifically, 233 μl of the reaction assay was mixed with 350 μl DNS-solution (10 g/l DNS, 2 g/l of phenol, 0.5 g/l of sodium sulphite and 10 g/l NaOH in ddH_2_O) and incubated for 15 min at 95°C. One hundred and seventeen microliter of Rochelles salt solution (400 g/l sodium tartrate in ddH_2_O) was added afterwards and the solution was cooled to RT for 15 min. The resulting solution was dispensed in triplicate to a 96 well plate and absorbance was measured at 540 nm. Substrate solution with xylose concentrations between 0.8 and 4 mM was used as a reference. The pH optimum was determined analogously with reactions using the same substrate in 50 mM citrate buffer (pH 5, 5.5, 6) or 50 mM NaPP (pH 6, 6.5, 7) at 38°C.

Combined activity of both enzymes was assayed by reactions of 5 g/l purified beechwood xylan (Megazyme) or wheat arabinoxylan (Megazyme) in 50 mM NaPP pH 6 at 32°C. The reaction was started by adding 5 μl enzyme solution (0.3–0.5 mg/ml) and 5 μl 50 mM NaPP pH 6 for single enzyme activity tests or 5 μl of each enzyme solution for combined activity tests. The reaction was stopped after 24 h by boiling at 95 °C for 10 min and analyzed by thin layer chromatography (TLC) and high-performance liquid chromatography (HPLC). TLC was performed using silica gel plates (Alugram SIL G, Macherey-Nagel GmbH & Co. KG). Ten microliter of sample was spotted onto the plate and developed in a chamber with chloroform/acetic acid/water 6:7:1 (v/v) as eluent solution. Afterwards, the plate was dried at RT and dyed by dipping in acetic acid/sulfuric acid/anisaldehyde 100:1:0.5 (v/v) solution followed by heating with a dryer until bands became visible. Additionally, samples were prepared for HPLC by centrifuging at 15.000 g for 10 min. Ten microliter of supernatant were injected to an Rezex ROAorganic acid H+(8%) Column from Phenomenex in a 1,100 Series System from Agilent Technologies with 5 mM sulfuric acid as eluent at a flow rate of 0.5 ml/min and a column temperature of 50°C. The resulting peaks were detected with a refractive index detector.

## Results

### Xylosidase Characterization

The highest score and e-value in the local BLASTp analysis for the xylosidase was obtained for ORY16049 against P49943, an xylosidase/arabinofuranosidase from *Bacteroides ovatus* (Whitehead, 1995), sharing 55.8% sequence identity. P49943 was described and characterized as BoXA (Jordan et al., 2017). In the following, ORY16049 will be called Xyl43Nc in reference to its putative activity and its organismal origin (*N. californiae*). Conserved domain search revealed that the protein consists mainly of a CoXyl43 like GH family 43 domain (311/327 amino acids). CoXyl43 is a calcium dependent xylosidase belonging to the GH 43 family (Matsuzawa et al., 2015). Xyl43Nc was further categorized as a member of GH 43_1 subfamily using dbCAN2. The highest confidence hits during structure prediction with Phyre2 were RS223-BX and CoXyl43. RS223-BX is another GH43 xylosidase that could also be activated by different ions (Lee et al., 2013) and shares similarity with CoXyl43 (Matsuzawa et al., 2017) and BoXA (Jordan et al., 2017).

**Figure 2:**
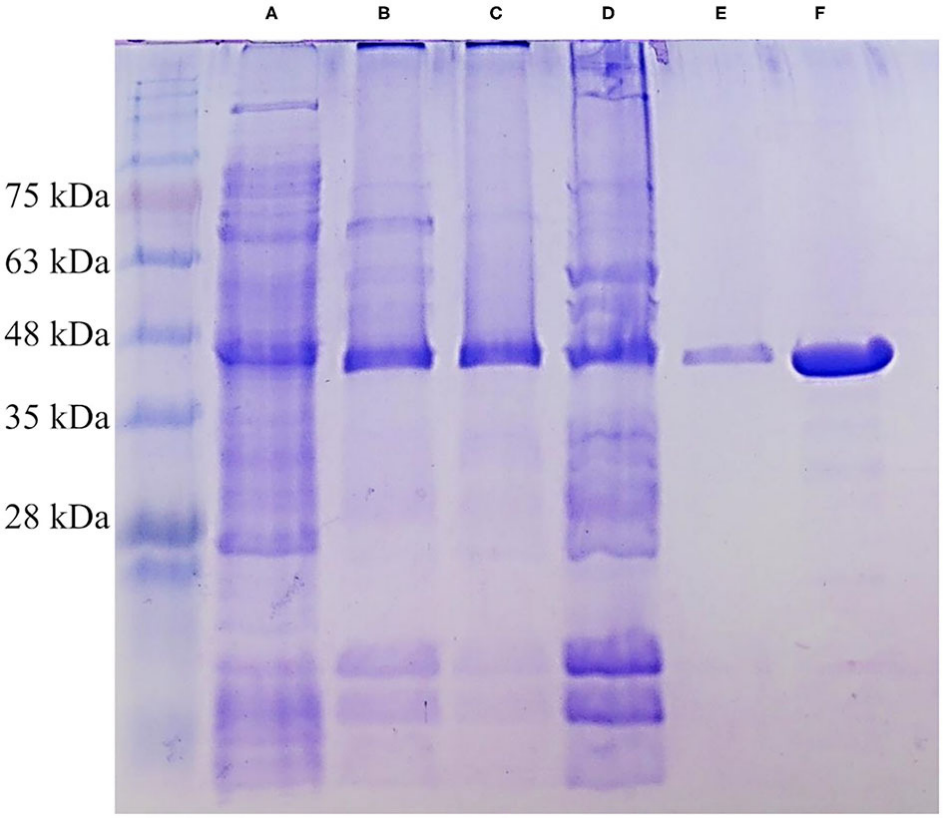
SDS-PAGE of different purification steps of Xyl43Nc. Crude protein extract **(A)**, affinity chromatography fraction 1 **(B)**, affinity chromatography fraction 2 **(C)**, pool of both fractions **(D)**, size exclusion chromatography **(E)** and concentrated size exclusion chromatography **(F)**. The strong band of Xyl43Nc between 35 and 48 kDa is visible in all samples. Marker: Bluestar Protein Ladder 10–180 kDa (Nippon Genetics).

**Figure 3:**
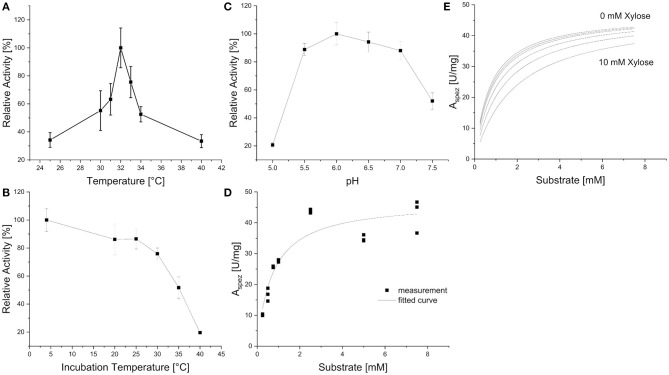
Biochemical characteristics of Xyl43Nc with 4-Nitrophenol-xylopyranoside as substrate. Activities are given in relation to the strongest activity at different reaction temperatures **(A)**, after incubation of the enzyme for 1 h at different temperatures **(B)** and at different pH values **(C)**. Reaction kinetics without product inhibition with measured specific activities and corresponding fitted curve **(D)**. Fitted reaction kinetics with different xylose concentrations between 0 and 10 mM **(E)**.

Xyl43Nc comprises 327 amino acids with an estimated molecular weight of 37.4 kDa. Due to the fusion with a 6xHis-Tag and a T7 epitope, the expressed protein has a calculated molecular weight of 39.3 kDa. On SDS-PAGE the purified enzyme was visible as a single band between 35 and 48 kDa in accordance to the predictions (Figure 2). Activity tests using the substrate 4-Nitrophenol-xylopyranoside, revealed a temperature optimum of 32°C for Xyl43Nc (Figure 3A). On both sides of the optimum, enzyme activity decreases rapidly. For instance, at 32 ± 2°C, enzyme activity decreased by ~ 50%. In terms of thermal stability, the enzyme was sensitive toward increased temperatures (Figure 3B). An incubation at 30°C for 1 h decreased the activity to 76%, while at 35°C the activity was decreased to 52% and at 40°C to 20% of the original activity. Similarly, high activity was detected between pH 5.5 and 7 with pH 6.0 being optimal (Figure 3C). Outside of this pH range activity decreased rapidly. Kinetic parameters were determined for the substrate 4-Nitrophenol-xylopyranosides (Figure 3D): K_m_ of 0.72 mM and k_cat_ of 29,28 s^−1^. The product xylose inhibited the reaction competitively (Figure 3E) with a K_i_ of 6.11 mM. In addition to xylosidase activity, a low arabinofuranosidase activity with 4-Nitrophenolarabinofuranosides as substrate was detected (K_m_ 0.1, k_cat_ 1.06 s^−1^).

In contrast to CoXyl43 and RS223-BX but similar to BoXA, Xyl43Nc activity seemed not to be dependent on metal ions. None of the tested ions (up to 10 mM) led to considerable increase in activity, while most were leading to a slight decrease in activity (Table 1) instead. The maximal decrease was observed with 1 mM Co (activity of 70.13%). This value had a high deviation and the higher Co concentration of 10 mM led to a smaller decrease of activity. EDTA also didn't have a great impact on the activity of the enzyme.

**Table 1:** Relative activity of Xyl43Nc toward 4-nitrophenol-xylopyranoside after addition of different ion concentrations or EDTA compared to the reaction without additives.

**Substance**	**Relative activity [%]**
Ba 10 mM	90.78 ± 4.63
Ba 1 mM	87.02 ± 1.93
Mg 10 mM	95.19 ± 2.92
Mg 1 mM	94.27 ± 3.94
Mn 10 mM	98.16 ± 1.73
Mn 1 mM	91.17 ± 5.36
Ni 10 mM	90.59 ± 4.19
Ni 1 mM	95.35 ± 2.03
K 10 mM	94.72 ± 1.21
K 1 mM	91.64 ± 7.09
Li 10 mM	88.53 ± 2.38
Li 1 mM	95.93 ± 5.15
Zn 10 mM	101.65 ± 7.32
Zn 1 mM	101.88 ± 3.80
Ca 10 mM	88.17 ± 1.24
Ca 1 mM	93.19 ± 4.58
Co 10 mM	88.22 ± 2.16
Co 1 mM	79.13 ± 10.48
Cu 10 mM	92.30 ± 3.01
Cu 1 mM	88.56 ± 2.21
10 mM EDTA	91.28 ± 9.51
20 mM EDTA	101.59 ± 16.33

### Phylogeny of Xyl43Nc

**Figure 4:**
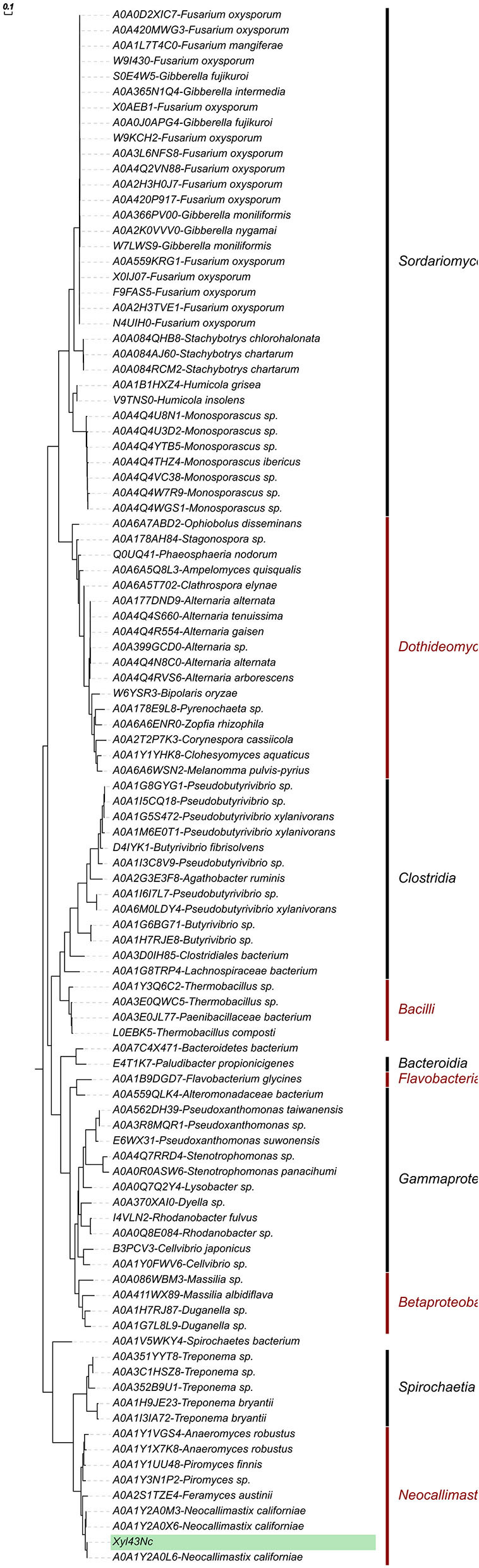
Phylogenetic relationship of Xyl43Nc to other GH43_1 enzymes. Sequences were aligned using T-Coffee (Notredame et al., 2000). Phylogenies were reconstructed using IQ-TREE v2.0.3 (Minh et al., 2020) with the automatic model selection (WAG+I+G4) and –bb 1,000. The resulting tree was rooted using MAD (Tria et al., 2017).

To study the evolutionary history of Xyl43Nc, one hundred and one homologous sequences, occurring only among bacteria and fungi, were identified and a maximum likelihood (ML) phylogeny was generated. The ML phylogenetic analysis revealed the presence of three strongly supported (bootstrap support range: 85–100%, Supplementary Figure 1) major branches (Figure 4). The first branch comprising the enzymes from *Ascomycota* (fungi) and the second the bacterial sequences from *Firmicutes, Bacteroides* and *Proteobacteria*. By contrast, Xyl43Nc clusters distinctly (branch support: 100%) alongside enzymes of other members of the phylum *Neocallimastigomycota* and those of the bacterial phylum *Spirochaetes*, suggesting a separate early evolutionary trajectory for GH43_1 of members of *Neocallimastigomycota* distinct from that of the fungal phylum *Ascomycota*. Interestingly, within the Xyl43Nc branch, A0AV5WKY4 from *Spirochaetes bacterium* formed a monophyletic clade (bootstrap support: 100%), which diverged earlier from the common ancestor of the rest of the members of *Spirochaetes* and *Neocallimastigomycota*, suggesting that GH43_1 of the latter, including Xyl43Nc, are likely to have been acquired via lateral gene transfer from *Spirochaetes* like enzymes. Horizontal gene transfer has been shown to have played a significant role in the evolution of anaerobic fungi (Haitjema et al., 2017; Murphy et al., 2019).

### Activity Against Natural Substrate

**Figure 5:**
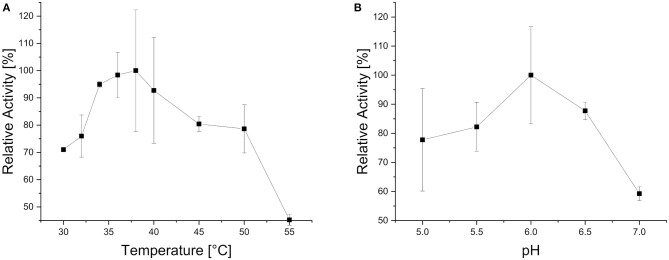
Biochemical characteristics of X11Nc when using purified beechwood xylan as substrate. Activities are given in relation to the strongest activity at different reaction temperatures **(A)** and at different reaction pH values **(B)**.

In order to test the activity of the novel xylosidase in a more natural context, endoxylanase activity was also needed. Therefore, local blast analysis of the *N. californiae* proteome was done against an endoxylanase database. The highest score was obtained for ORY50654 against B8YG19, published as XynS20E (Pai et al., 2010), from *Neocallimastix patriciarum* with 87.44% identity. ORY50654, renamed X11Nc, has a length of 655 amino acids and conserved domain search detected a GH11 domain (178 amino acids), an alpha/beta-hydrolase super family domain (265 amino acids) and two CBM_10 dockerin domains (38 and 35 amino acids). Conserved domain search of XynS20E yielded in the same quantity and type of domains as for X11Nc. The search results are in accordance to the previously published results describing endoxylanase GH11, acetylesterase and two dockerin domains (Pai et al., 2010). X11Nc has an estimated molecular weight of 70.7 kDa and the expressed version of the protein including tags results in a molecular weight of 72.6 kDa, which can be detected on the SDS PAGE between the 63 kDa and 75 kDa marker bands. X11Nc has a temperature optimum of 38°C (Figure 5A) and a pH optimum of 6 (Figure 5B).

**Figure 6:**
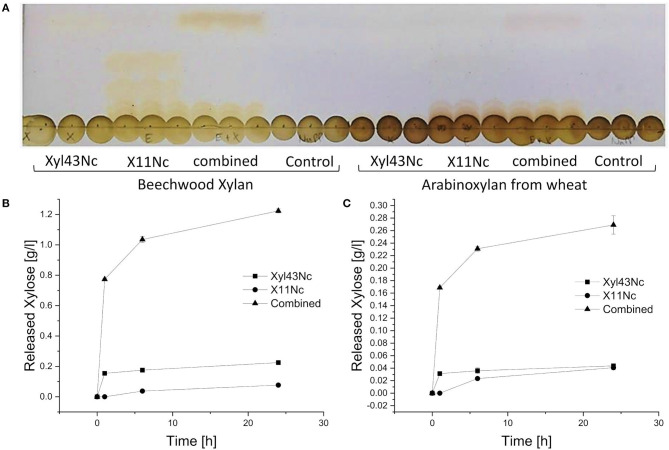
Individual and combined reactions of Xyl43Nc and X11Nc with purified beechwood xylan and arabinoxylan from wheat as substrates. Thin layer chromatography of the triplicate reactions after 24 h **(A)**. Slight bands of xylose can be seen in the reactions of Xyl43Nc alone and much stronger xylose bands in the combined reactions. Concentrations of released xylose during the reactions with purified beechwood xylan **(B)** and arabinoxylan from wheat **(C)**.

To evaluate synergistic effects, X11Nc and Xyl43Nc were used individually as well as in combination against beechwood xylan and arabinoxylan from wheat. When Xyl43Nc was used alone, only weak activity was observed against beechwood xylan (Figure 6A), whereas the single application of the endoxylanase X11Nc seemed to release polysaccharides of different length. When both enzymes were combined, released oligo-saccharides from the endoxylanase reaction disappeared, substituted by a strong xylose band instead. The amount of released xylose seemed to be higher in the combined reaction compared to the reaction of the xylosidase alone. Longer saccharides from the endoxylanase reaction seemed to remain untouched by the xylosidase activity implying a low or inexistent activity toward them. The activity of the enzymes on arabinoxylan from wheat seemed to follow the same rules but with much fainter saccharide spots. The HPLC analysis confirmed these results. The highest amount of xylose was released after 24 h by the combined action reaching 1.22 g/l on beechwood xylan and 0.26 g/l xylose on arabinoxylan from wheat (Figure 6B). Under the same conditions Xyl43Nc alone just released 0.22 g/l on beechwood xylan. On arabinoxylan from wheat the amount of released xylose by Xyl43Nc was calculated to be 0.04 g/l, which is already below the calibration lower limit (Figure 6C). Despite the low activity against 4-Nitrophenolarabinofuranosides described above, no release of arabinose from the reactions with arabinoxylan could be detected. In all reactions with arabinoxylan from wheat as substrate the amount of released xylose was considerably lower than from beechwood xylan. The HPLC results revealed a strong decrease in activity after the first hour of reaction for both the combined reaction and the reaction of Xyl43Nc alone. Specifically, the combined reaction on beechwood xylan released 0.77 g/l xylose during the first hour, an additional 0.27 g/l during the following 5 h but only 0.19 g/l within another 18 h of reaction time. This decrease in activity is in accordance with the temperature instability of Xyl43Nc described above.

## Discussion

GH43 enzymes have been detected in anaerobic fungi previously (Youssef et al., 2013; Couger et al., 2015; Henske et al., 2017; Hagen et al., 2020) but none has been kinetically and thermodynamically characterized to the best of our knowledge. Xyl43Nc showed a very low temperature optimum at 32°C when compared to the growth optimum of 39°C for anaerobic fungi (Theodorou et al., 2005). The latter temperature has a clear negative impact on the activity of Xyl43Nc xylosidase (Figures 3B, 6B,C). Glycosylation has been shown to increase the stability and activity of other proteins (Bonzom et al., 2019), raising the question of our data being influenced by choosing *E. coli* as expression host. However, the only other heterologously expressed xylosidase from anaerobic fungi was also expressed in *E. coli* and had a temperature optimum of 39°C (Morrison et al., 2016a) in accordance to the culture temperature. Future studies will have to reveal potential influence of the host system on the production of anaerobic fungal CAZymes. Another possibility for the lack of stability of Xyl42Nc at the culture temperature of *N. californiae* would be the natural occurrence in a protein complex which increases the final stability of the enzyme. These cellulosome named complexes are widely spread throughout the *Neocallimastigomycota* phylum (Haitjema et al., 2017). Enzymes occurring in cellulosomes contain a dockerin domain which allows them to bind to the complex (Haitjema et al., 2017). Conserved domain search did not identify dockerin domains in Xyl43Nc making the absence of a naturally occurring binding partner less likely for the unexpected temperature behavior of the enzyme. Consequently the addition of such a domain to Xyl43Nc could provide a more stable and even active enzyme, as cellulosomes have increased biomass degradability through synergistic enzyme activity and targeting of the substrate through carbohydrate binding modules (Gilmore et al., 2015). Recently a chimeric enzyme consisting of GH5 from *Thermatoga maritima* and a dockerin domain from *Piromyces finnis* was shown to be recruited into native cellulosomes highlighting the potential for the construction of full artificial cellulosomes (Gilmore et al., 2020).

The only biochemically characterized xylosidase from *Neocallimastigomycota* is Bgxg1, a multifunctional GH39 encompassing xylosidase, galactosidase and glucosidase activities (Morrison et al., 2016a). The properties for Bgxg1 and other xylosidases are listed in Table 2. When compared to Xyl43Nc, Bgxg1 had a similar pH optimum but was more stable at conditions differing from the optimum for both temperature and pH. Although the turnover number k_cat_ for Bgxg1 was not calculated, it had a lower K_m_ and a higher V_max_ than Xyl43Nc. The only other anaerobic fungal xylosidases with evidence at protein level were purified from *Neocallimastix frontalis* (Hebraud and Fevre, 1990; Garcia-Campayo and Wood, 1993). Both enzymes showed a similar pH (6.4 and 6.5) and temperature optima (35 and 37°C). When comparing Xyl43Nc to the related xylosidase BoXA from *Bacteroides ovatus* the enzyme shows a lower K_m_ and a higher k_cat_ determined for the artificial substrate 4-Nitrophenol-xylopyranosides. The activity of xylosidases on 4-Nitrophenol-xylopyranosides doesn't necessarily correlate with the activity on natural substrates like xylobiose with both higher and lower activities being possible (Jordan and Wagschal, 2010). BoXA had an k_cat_ of 69 s^−1^ for xylobiose and of 7.82 s^−1^ for 4-Nitrophenol-xylopyranosides (Jordan et al., 2017; Table 2). Further characterization of Xyl43Nc with xylooligosaccharides is needed to determine the activity of the enzyme against its natural substrate and finally evaluating the enzymes potential for lignocellulose degradation. Up to date the highest xylosidase activity has been reported for Wxyn43 with a k_cat_ of 258 s^−1^ on 4-Nitrophenol-xylopyranosides and a k_cat_ of 961 s^−1^ on xylobiose (Falck et al., 2016).

**Table 2:** Biochemical properties of selected xylosidases on the substrates 4-nitrophenol-xylopyranoside (NPX), xylobiose (X2), xylotriose (X3) and xylotetraose (X4).

**GH**	**Enzyme**	**Substrate**	**pH**	**Temp. [^**°**^C]**	**K_**m**_ [mM]**	**V_**max**_ [U/mg]**	**k_**cat**_ [s-1]**	**References**
39	Bgxg1	NPX	6	39	0.00485	127	-	
								Morrison et al., 2016a
43	Xyl43Nc	NPX	6	32	0.72	46.97	29.28	Here
43	BoXA	NPX	6	25	4.57	-	7.82	
								Jordan et al., 2017
		X2	6	25	0.606	-	69	
		X3	6	25	0.0908	-	19.1	
		X4	6	25	0.124	-	25.9	
43	Wxyn43	NPX	6	37	7.4	-	258	
								Falck et al., 2016
		X2	6	37	7.2	-	961	
		X3	6	37	6.5	-	900	
		X4	6	37	17	-	770	

The pH optimum of endoxylanase X11Nc was in the same range as reported for the closely related endoxylanase XynS20E (Pai et al., 2010), but the temperature optimum was much lower (38 vs. 49°C). The combined reaction of Xyl43Nc xylosidase and X11Nc endoxylanase indicated that the xylosidase was able to hydrolyze smaller oligosaccharides resulting in xylose release. This tendency to shorter substrates is a common feature of xylosidases (Polizeli et al., 2005). The reactions with arabinoxylan as substrate revealed the same pattern as the reactions with beechwood xylan but resulted in lower xylose concentrations. The complete degradation of arabinoxylan requires removal of arabinose substitutions by an arabinofuranosidase because they can inhibit xylanase activity sterically (Pollet et al., 2010) leading to the observed lower activity. Although Xyl43Nc exhibited a low arabinofuranosidase activity against 4-Nitrophenolarabinofuranosides, release of arabinose from arabinoxylan was not detected, suggesting a negligible activity for the enzyme against natural substrates.

Interestingly the highest ranked enzymes in the conserved domain search and the highest ranked to be used as model for the structure of Xyl43Nc by Phyre2 are all highly ion dependent. In contrast, Xyl43Nc wasn't further activated by the addition of different ions and addition of the chelating agent EDTA didn't decrease enzyme activity. This is in accordance with BoXA, which also shares 80.6% identical residues with RS223-BX in general and 19/20 residues of the active site (Jordan et al., 2017) but is ion independent despite high overall similarity (Jordan et al., 2018). Both enzymes also share high identity in the active site with CoaXyl43 (Jordan et al., 2018). The main difference in the active center between the ion dependent xylosidases RS223-BX and CoaXyl43 is the coordination of the calcium ion. RS223-BX coordinates the ion by His-274 and Asp-85 (Jordan et al., 2015). While the first amino acid is conserved in CoaXyl43 (His-319) the latter is substituted by Ala-126 (Matsuzawa et al., 2017). We suppose that during structure prediction the algorithm selected RS223-BX and CoaXyl43 as models because of the lack of a crystal structure of BoXA or a similar enzyme. Xyl43Nc is ion independent in contrast to these models and therefore no tertiary structure could be determined. As proposed before (Jordan et al., 2018) a crystal structure of BoXA could elucidate the reaction mechanisms of this type of ion independent xylosidase. The exact catalytic mechanism of ion independent GH43 xylosidases could be of fundamental interest for future methods of enzymatic xylan degradation.

We investigated the evolution of Xyl43Nc along with similar enzymes from other fungi and bacteria. Our results were consistent with previous studies in highlighting the role of horizontal gene transfer in the evolution of *Neocallimastigomycota* (Haitjema et al., 2017; Murphy et al., 2019). Xyl43Nc and similar proteins from anaerobic fungi cluster with *Spirochaetes* and the phylogeny suggests a common ancestry and horizontal gene transfer from these bacteria to *Neocallimastigomycota*. Previous studies have identified *Proteobacteria, Bacteroidetes, Firmicutes, Spirochaetes* as the main bacterial horizontal gene transfer donors for anaerobic fungi (Murphy et al., 2019). Outside of the phylum *Neocallimastigomycota*, the evidence for gene transfer of GH from bacteria to fungi is limited (Lange et al., 2019).

In conclusion, we characterized the first GH43 xylosidase from *Neocallimastigomycota* starting to reveal the untapped xylan degrading resource of this fungal phylum and confirming its potential for future exploitation. Over the last years more genomic and transcriptomic data has become available for various strains of the phylum (Solomon et al., 2016; Haitjema et al., 2017; Wilken et al., 2021). Transcriptomic data could guide the search for potential CAZyme candidates for expression through identifying the enzyme/gene variants which are expressed under certain cultivation conditions. Although the information on the biochemical properties of anaerobic fungal enzymes are limited, there have been promising results (Morrison et al., 2016a,b) highlighting the necessity and relevance of biochemical characterization studies. Future efforts should also investigate the influence of the expression system on the activity and stability of heterologous *Neocallimastigomycota* enzymes. Developing novel expression systems for *Neocallimastigomycota* enzymes could open the way for a more extended heterologous production and characterization of these enzymes. The highly active enzymes of this phylum could promote biomass pretreatment and thereby pave the way toward a bio-based economy.

## Data Availability Statement

The datasets presented in this study can be found in online repositories. The names of the repository/repositories and accession number(s) can be found in the article/Supplementary Material.

## Author Contributions

MS conceptualized the study and wrote the original draft. MS, JH, ZH, and HA performed the experiments and analyzed the data. HA and KO reviewed and edited the draft and constructively contributed to the content. All authors have read and agreed to the published version of the manuscript.

## Conflict of Interest

The authors declare that the research was conducted in the absence of any commercial or financial relationships that could be construed as a potential conflict of interest.
