# Supplementary material for: Characterization and Phylogenetic Analysis of a Novel GH43 β-Xylosidase From Neocallimastix californiae
Source: Front Fungal Biol. 2021 Jul 7;2:692804. doi: 10.3389/ffunb.2021.692804 (PMC10512374; doi:10.3389/ffunb.2021.692804)
Supplement: Supplementary file 1 [file Data_Sheet_1.DOCX]

Supplementary Material

Supplementary Figure 1. Phylogenetic relationship of Xyl43Nc to other GH43_1 enzymes with bootstrap values. Sequences were aligned using T-Coffee (Notredame et al., 2000). Phylogenies were reconstructed using IQ-TREE v2.0.3 (Minh et al., 2020) with the automatic model selection (WAG+I+G4) and –bb 1000. The resulting tree was rooted on midpoint.


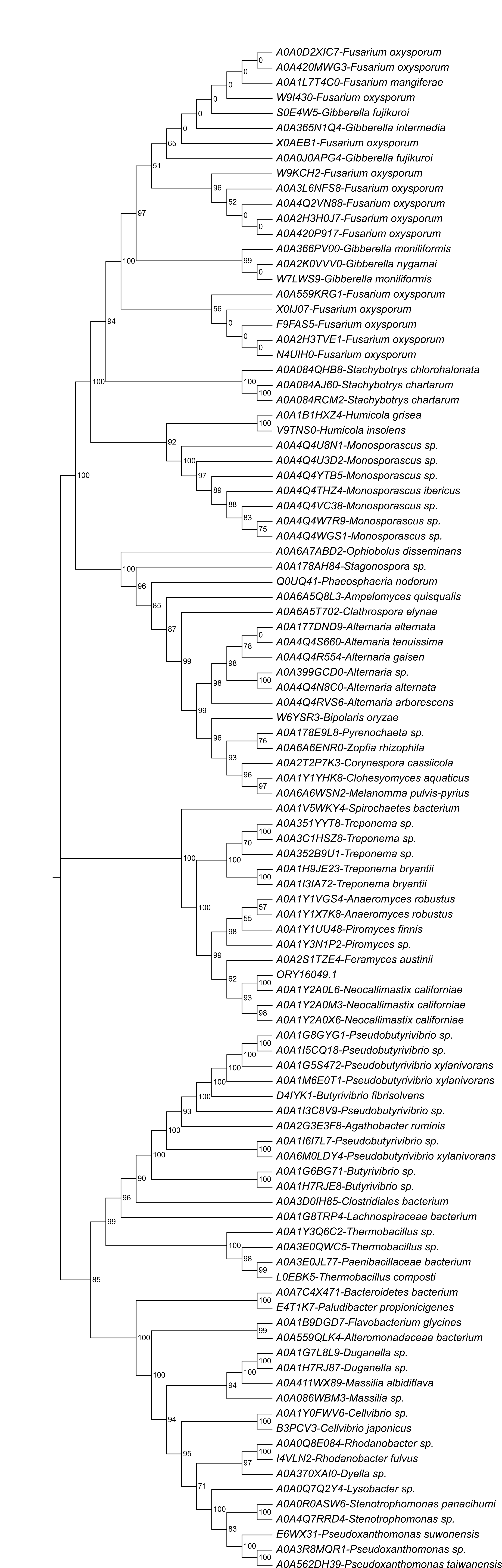


Supplementary 2. DNA sequence of Xyl43Nc as used for expression

ATGGTAACCATCGAGAAAAGGCCTTTGGTAACAGATACTTACACGGCGGATCCCAGTGCACACGTTTTTAATGGTAAAATCTACATTTATCCTAGCCATGATCGGGACATCGAGACTATCGACAACGATAACGGAGACCAGTATGATATGAAGGATTATCACGTATACGAGATGGACGATGAGAACACACTGCCTCGCGACTGCGGCAAGGTTTTGGATATCAAGGATATTCCGTGGGTCTCTAAACAATTGTGGGCTCCGGACTGCGTTGAAAAAGACGGGAAGTATTATTTTTTCTACCCCGCAAGAGACAAAGAAGGCTTCTTCAGAATAGGGGTGGCGATAGGAGATAAACCTGGCGGACCGTTCAAGCCGGAGCCCAACTACATCCAGGGAACTTACTCGATAGATCCTTGCATCTTTCCTGACACCGACAAGAATTTCTACCTCACCTTTGGAGGCATCTGGGGTGGCCAGCTTGATAAATATAGAAATAACGTTTACGACGAGAAAAACGAGGAACCCACAGGAGAGACCCCTGCAGTGTGCGCTAAGATAGCCAAGATGAACCCTGACATGAAAACGTTAGCAGAAGCACCGCGCGATATTGTGATACTCGATGAAAATGGCAAACCCCTTACAGGGAAGGACCACGACCGACGTTATTTTGAAGACCCATGGCTATATAAAAAGGGCGACACGTACTATTTTACATACTCGACCGGTGACACGCACTTCTTGTGCTGGTCCACTAGCAAGAATGTCTATGGGCCGTATACGTATGGAGGAAAAATATTAACACCCGTGTTGGGGTGGACAACCCACCATTCCATCCTAGAGTACCACGGAAAATGGTGGCTGTTTTACCACGATTGCGAAATTAGTAAGGGTGTTAATCATAAGCGGAACGTGAAGTTCCGGGAGCTGAAGTACGACGACAAGGGCGGTATTATCACAATGGACGGTACCCTAGGGGCA

Supplementary 2. DNA sequence of X11Nc as used for expression

ATGAGACTGGGAATTGCGTTAAGTACCATCGCAGTATTGCTGACTGCTACAAGTGCTAGAAATTTGGACAAGAGACAATGGGGTTGGGGGGGTTTTGGTGGCGGTGGAAATGGTGGTGGTAAGACTATAAATGATTATAAACGTGAGCAAGTTAGCGGTCGTGATATCCACGTTTACGCCCCGTCAAATCTTGCCCCAAACTCACCTCTTCTGTTGTCCTTACATGGCATGGATCAGGATCCAAATTATCAACAATCAAATACTCATTGGGAAACATTGGCCGATAAAGAAGGATTTGTAGTAGTTTATCCCAGAGGAGGAACTGGAATGAGCACTTGGGACATTCAAGGTACCAAAGACACTCAATGGGTTTCACAGATTATCGACCAAATGAAGAAAGAATACAATATAGATACTAAACGTGTTTATCTTTCTGGGTTTAGTATGGGAGGAATGTTCACATATCATGCAATGTCACAAATTGCAAACAAAATTGCAGCCTTCGCCCCGTGTTCCGGTCCGAATGTTTTCGGTGCCTCCAAGGCTCAGCGTCCAGTTCCTATCTTCCATGTTCATGGCACTAACGATGATGTTCTAAACTATAACCAGGTGGAGGGGTTCCTGAAGAATTACCGAGATCAGTTCCACTGCCCCTCACAAGCTGACACTAAGACAAACTACCCAAACAGAGAGAATCCTAATGCAACCCTGTATAGCTGGGGTCCATGCGATAAAGGGGTTTACATTAAACATTTAAAATTGCAAGGGAGAGGCCACTCCCCTTCTAGTGCGGACATTGAGGACATCTGGAACTTTCTGAAGGAATACACTGTTGATGGACCAGTCTCAGCTAGCGGTAATACCAACCCAACAACAGGAGGTAACGGCGGTAACGGAGGCAATGGTGGTAACGGTGGAAACGGTAATTCAAACGCGAAGTGTAGTTCAAATATAACTAAACAGGGGTATAAGTGCTGTTCGTCTAACTGTGAAGTGGTCTACACTGACACCGACGGAGACTGGGGTGTTGAAAATGACCAATGGTGTGGATGTGGAAACAGAGTCACTGTTGGAAATGGGACCTGTTCAGCTAAGATTACCTCCCAGGGATATAAGTGTTGCCCAAACGGTTGTATCATTTATTATACTGACGATGATGGTACTTGGGGTGTTAACAACGACGAGTGGTGTGGTTGCGGAGGCGGATCTAATAACGGCCAATCAAACGGCGGCTCTAGCTCCAATGGTGGTTCTTCCTCCAATGGTGGTTCTTATCAAGGTGCCGGCAATACAAATTTCTGCTCTAATGCCAAGCACTCGGGTAAGTCCGAAAAAGTAACCTCCAACAAAGTTGGTTCGATTAATGGGATAGGTTACGAGCTTTGGTCAGACTCTGGTAACAATTCAGCAACTTTCTATGAGGACGGATCGTTCTCCTGTTCATTTCAGTACGCTAAAGACTACCTATGTAGATCGGGTCTGTCATTTGACTCCACCAAAACTCACCAACAGATCGGACATATATACGCTGAATTTAAATTAGTCAAGCAAAACATTCAGAACGTTGATTATTCTTACGTTGGAATATATGGCTGGACACGAAACCCTTTAGTTGAATTTTACGTTGTTGATAACTGGTTGTCTCAATATCGGCCTGGAGACTGGGTTGGGAACAAAAAACACGGAGATTTCACTATTGACGGAGCCAAGTACACAGTGTATGAAAATACAAGATATGGCCCTAGTATAGATGGAAACACCAACTTCAAACAATACTTCTCAATAAGACAACAGCCTAGGGACTGTGGTACTATAGATATTACTGCACATTTTCAACAATGGGAGAAATTAGGGATGACTATGGGTAAAATGCATGAGGCTAAAGTGTTGGGAGAGGCAGGTTCAAACGGAGGAGGTACGTCTGGTACCGCCGATTTTCCATATGCGAAAGTTTACGTTAAGAAT
